# A phase I study of the SRC kinase inhibitor dasatinib with trastuzumab and paclitaxel as first line therapy for patients with HER2-overexpressing advanced breast cancer. GEICAM/2010-04 study

**DOI:** 10.18632/oncotarget.17113

**Published:** 2017-04-14

**Authors:** Alberto Ocana, Marta Gil-Martin, Miguel Martín, Federico Rojo, Silvia Antolín, Ángel Guerrero, José Manuel Trigo, Montse Muñoz, Atanasio Pandiella, Núria Gonzalo Diego, Susana Bezares, Rosalía Caballero, Eva Carrasco, Ander Urruticoechea

**Affiliations:** ^1^ Department of Medical Oncology, Complejo Hospitalario Universitario de Albacete, Albacete, Spain; ^2^ Department of Medical Oncology, Institut Català d'Oncologia-IDIBELL, L'Hospitalet, Barcelona, Spain; ^3^ Department of Medical Oncology, Instituto de Investigación Sanitaria Gregorio Marañón, Universidad Complutense, Madrid, Spain; ^4^ Fundación Jiménez Díaz, Madrid, Spain; ^5^ Department of Medical Oncology, Complejo Hospitalario Universitario A Coruña, La Coruña, Spain; ^6^ Department of Medical Oncology, Instituto Valenciano de Oncología, València, Spain; ^7^ Department of Medical Oncology, Hospital Clínico Universitario Virgen de la Victoria, Málaga, Spain; ^8^ Department of Medical Oncology, Hospital Clinic i Provincial de Barcelona, Barcelona, Spain; ^9^ Centro de Investigación del Cáncer and CIBERONC, CSIC-Universidad de Salamanca, Salamanca, Spain; ^10^ Pharmacokinetics Laboratory, Farmacia ICO Metropolitana, IDIBELL, Instituto Catalán de Oncología, Hospital Duran i Reynals, Barcelona, Spain; ^11^ Scientific Department, GEICAM Spanish Breast Cancer Group, Spain; ^12^ Translational Department, GEICAM Spanish Breast Cancer Group, Spain; ^13^ Department of Medical Oncology, Fundación Onkologikoa, Donostia, Gipuzkoa, Spain

**Keywords:** HER2 positive breast cancer, dasatinib, SRC kinase, trastuzumab resistance, metastatic breast cancer

## Abstract

The anti-HER2 antibody trastuzumab have shown clinical activity in combination with chemotherapy in different breast cancer settings. However, most of patients treated with this antibody progress after a period of treatment. Activation of the kinase SRC has been linked with resistance to trastuzumab in several preclinical studies. We designed a phase I clinical study to explore the activity of weekly trastuzumab (2 mg/kg) plus paclitaxel (80 mg/m^2^) in combination with the anti-SRC kinase inhibitor Dasatinib in the first line treatment of HER2 metastatic breast cancer. The primary objective was to determine the maximum tolerated dose (MTD) and recommended phase II dose (RP2D); secondary objectives included efficacy, objective response rate (ORR), pharmacokinetics and pharmacodynamics. A “3+3” design guided dose escalation with two oral dose levels of dasatinib: 100mg (DL1) and 140 mg (DL2). 10 patients were included in the phase I part. Dasatinib 100 mg q.d. was established as the recommended RP2D. The median number of administered cycles was 12 (range, 1 to 18). Grade 3 treatment-related AEs at DL1 were diarrhea (*n* = 2), hyponatremia (*n* = 1), fatigue (*n* = 1), and AST/ALT elevation (*n* = 1). *A* significant reduction in p-SRC expression on epidermal keratinocytes on sequential skin biopsies was observed. In conclusion, we describe the feasibility of the combination of dasatinib, trastuzumab and paclitaxel, and its effect on proteins involved in trastuzumab resistance. The phase II part of this study is currently evaluating efficacy.

## INTRODUCTION

Personalized medicine refers to the identification of druggable molecular vulnerabilities in specific tumors by using companion diagnostics, that therefore permits the selection of patients based on the presence of a biomarker [1, 2]. An example has been the development of anti-HER2 therapies for HER2-overexpressing cancers [3]. The monoclonal antibody trastuzumab was designed to target the transmembrane tyrosine kinase receptor HER2 in tumors that overexpressed this protein due to gene amplification [4]. From a clinical perspective, the addition of this antibody to chemotherapy showed an increase in survival in breast cancer patients, both in the metastatic and adjuvant settings [5, 6].

Although no uncertainty exists about the clinical benefit provided by trastuzumab, the majority of metastatic patients with HER2 overexpressing tumors treated with this compound progress after a few months of treatment [3]. In this clinical scenario, novel agents have been incorporated to the armamentarium of breast cancer patients with HER2 positive tumors, including the kinase inhibitor lapatinib, the anti-HER2 antibody pertuzumab or the antibody drug conjugate T-DM1 [7–9].

Several mechanisms linked with trastuzumab resistance have been described including activation of membrane receptors like the insulin-like growth factor I receptor (IGF-1R) [10], or MET (also called hepatocyte growth factor receptor, HGFR) [11]. Others comprise intrinsic mechanisms of the receptor itself, like the overexpression of the heterodimeric glycoprotein complex Muc4/SMC [12] or the expression of activated C-terminal truncated forms of the receptor [13]. Activation of downstream mediators like the PI3K-mTOR pathway by loss of PTEN or potentially by other phosphatases like INPP4B and PP2A [14]; or mutations at the PI3KCA gene [15, 16] have been suggested as associated with resistance, in addition to other mechanisms like down-regulation of p27 [17] or cyclin E overexpression [18].

Taking into consideration that some of these mechanisms have not been confirmed or reproduced in clinical studies [19, 20], and that the incidence of resistance to anti-HER2 therapies is significantly higher than the frequency of these molecular abnormalities, it is suggested that alternative mechanisms of resistance must exist. One of such mechanisms includes the activation of the cytosolic kinase SRC. Three different studies have shown the key role of SRC inhibition to augment trastuzumab activity. It was observed that SRC was activated in both acquired and the novo trastuzumab resistance cells and in breast cancer patients that progressed to trastuzumab [21]. In another study, the authors identified SRC as a mediator of trastuzumab resistance by performing a tyrosine phosphoproteome of sensitive and resistant cells using immunoaffinity-enriched mass spectrometry [22]. Finally, in our experience using preclinical models of HER2-overexpressing breast tumors, the administration of the multi-tyrosine kinase inhibitor dasatinib with trastuzumab increased the activity of this antibody in a synergistic manner. The mechanism of action of the combination included the induction of DNA double-strand breaks, cell cycle arrest, and caspase-independent apoptosis [23].

Based on these preclinical data, GEICAM, the Spanish Breast Cancer Group, designed a phase I-II trial to evaluate the safety and clinical activity of the association of dasatinib to a standard treatment with trastuzumab and paclitaxel. Pharmacodynamic analyses were included to corroborate findings identified in preclinical models that could help to the clinical development of this combination. In this article, we report the safety and pharmacokinetics data from the phase I part of the study, and results from the exploratory biomarker analyses.

## RESULTS

### Patient characteristics

Between Aug 2011 and April 2013, ten patients were enrolled in six Spanish centers, receiving at least one cycle of the study treatment combination. Patients’ characteristics are described by dose level in Table 1. Median age was 48 years (range, 35-81), hormone receptor positive (estrogen receptor (ER) positive +/− progesterone receptor (PgR) positive) tumors were present in 70% (*n* = 7) of patients; and 50% (*n* = 5) were postmenopausal. Forty percent (*n* = 4) of patients received previous trastuzumab in the neoadjuvant and/or adjuvant settings and half of the patients received previous hormone therapy.

**Table 1:** Patients’ characteristics by dose levels

Characteristics	Dasatinib Dose Level (mg)		
	Dose Level 1 100 (*n* = 6)	Dose Level 2 140 (*n*= 4)	All patients (*n*= 10)
Age, years (median; range)	44 (35-81)	49 (47-73)	48 (35- 81)
Race, *n* (%)			
Caucasian	5 (83)	4 (100)	9 (90)
Hispanic	1 (17)	0 (0)	1 (10)
ECOG PS, *n* (%)			
0	2 (33)	3 (75)	5 (50)
1	4 (67)	1 (25)	5 (50)
Menopausal status, *n* (%)			
Premenopausal	4 (67)	1 (25)	5 (50)
Postmenopausal	2 (33)	3 (75)	5 (50)
Hormone receptors, *n* (%)			
ER+ / PgR+	4 (67)	1 (25)	5 (50)
ER+ / PgR-	1 (17)	1 (25)	2 (20)
ER- / PgR-	1 (16)	2 (50)	3 (30)
Previous trastuzumab treatment			
Yes	2 (33)	2 (50)	4 (40)
No	4 (67)	2 (50)	6 (60)
Previous hormonotherapy treatment			
Yes	4 (67)	1 (25)	5 (50)
No	2 (33)	3 (75)	5 (50)
Previous radiotherapy treatment			
Yes	1 (17)	2 (50)	3 (30)
No	5 (83)	2(50)	7 (70)
Other previous chemotherapy or biological treatment			
Yes	2 (33)	2 (50)	4 (40)
No	4 (67)	2 (50)	6 (60)

mg: milligram; n: number of patients; ECOG: Eastern Cooperative Oncology Group; PS: performance status; ER: estrogen receptor; PgR: progesterone receptor.

### Dose escalation and determination of RP2D

Six patients were treated at DL1 (100 mg) and four at DL2 (140 mg). The median number of administered cycles was 12 for DL1 (range, 1 to 18) and 3.5 cycles for DL2 (range, 1 to 8 cycles) (see Table 2). The median relative dose intensity (RDI) of dasatinib differed between both dose levels being of 100% at DL1 and 72% at DL2. Of the first three patients included in DL1, one experienced a DLT consisting on grade 3 transaminase elevations; three additional patients were recruited at this dose level without any new DLT reported. At DL2, two out of four patients experienced a DLT, one consisting on grade 3 pneumonitis, and the other on grade 3 nausea/vomiting not controlled with adequate supportive therapy. All DLTs were reversible. The main reasons for treatment discontinuation were AEs and investigator's decision (*n* = 4, 40%, each); two patients discontinued due to disease progression (*n* = 2, 20%). AEs causing treatment discontinuation at DL1 were grade 3 transaminase elevations (DLT) and grade 3 diarrhea, and at DL2 grade 3 pneumonitis (DLT) and grade 2 paresthesia.

**Table 2:** Maximum number of cycles received per patient and by Dasatinib dose level

Maximum number of cycles received per patient	Number of patients	
	Level 1 100 mg (*n*=6)	Level 2 140 mg (*n*=4)
1 cycle	1	1
2 cycles	-	1
5 cycles	-	1
8 cycles	1	1
10 cycles	1	-
14 cycles	1	-
16 cycles	1	-
18 cycles	1	-
Median number of cycles (range)	12 (1-18)	3.5 (1-8)

mg: milligram; n: number of patients.

Dasatinib 100 mg q.d. plus weekly paclitaxel 80 mg/m^2^ plus weekly trastuzumab 2 mg/kg was established as the recommended phase 2 dose (RP2D). Additional patients have been included in the phase II part of this study. Results of such study will be reported shortly.

### Safety

There was not any grade 4 AE or serious AE reported. Grade 3 treatment-related AEs at DL1 were diarrhea (*n* = 2), hyponatremia (*n* = 1), fatigue (*n* = 1), and AST/ALT elevation (*n* = 1) whereas at DL2, nausea, vomiting, abdominal pain, hyponatremia and pneumonitis (*n* = 1, for each individual AE). One patient had grade 2 left ventricular ejection fraction reduction at DL1. Table 3 shows in detail additional data for grade 1-2 AEs at both dose levels.

**Table 3:** Treatment related adverse events by dose level (NCI-CTCAE v4.03)

AEs	Dasatinib Dose Level (mg)					
	Level 1 (*n*= 6)			Level 2 (*n*= 4)		
	*n* (%)			*n* (%)		
	G1	G2	G3	G1	G2	G3
Red blood cells count decreased	5 (83)	1 (17)	-	2 (50)	-	-
Leukopenia	3 (50)	1 (17)	-	2 (50)	1 (25)	-
Neutropenia	4 (67)	-	-	1 (25)	1 (25)	-
AST increase	3 (50)	-	1 (17)	4 (100)	-	-
ALT increase	4 (67)	1 (17)	1 (17)	3 (75)	-	-
GGT increase	1 (17)	1 (17)	-	-	-	-
Hypocalcemia	1 (17)	2 (33)	-	1 (25)	1 (25)	-
Hyponatremia	1 (17)	-	1 (17)	-	-	1 (25)
Nausea	3 (50)	-	-	-	2 (50)	1 (25)
Vomiting	2 (33)	-	-	-	-	1 (25)
Diarrhea	-	2 (33)	2 (33)	1 (25)	1 (25)	-
Abdominal pain	1 (17)	-	-	-	-	1 (25)
Oral mucositis	3 (50)	-	-	-	-	-
Dyspepsia	2 (33)	-	-	-	-	-
Dysgeusia	1 (17)	-	-	-	1 (25)	-
Anorexia	3 (50)	-	-	-	2 (50)	-
Alopecia	1 (17)	4 (67)	-	1 (25)	-	-
Fatigue	3 (50)	-	1 (17)	-	3 (75)	-
Pneumonitis	-	-	-	-	-	1 (25)
Peripheral sensory neuropathy	3 (50)	-	-	1 (25)	-	-
Arthralgia	2 (33)	1 (17)	-	1 (25)	-	-
Myalgia	1 (17)	1 (17)	-	-	-	-
LVEF decrease	-	1 (17)	-	-	-	-
Pericardial effusion	-	-	-	-	1 (25)	-

NCI-CTCAE: National Cancer Institute-Common Terminology Criteria for Adverse Events; mg: milligram; n: number of patients; AE: adverse event; G: grade; AST: aspartate aminotransferase; ALT: alanine aminotransferase; GGT: gamma-glutamyl transpeptidase; LVEF: left ventricular ejection fraction.

At DL1, there were no dose reductions for any of the study drugs. At DL2 there were four dasatinib dose reductions, two due to grade 3 abdominal pain, and two due to grade 3 nausea and vomiting and grade 2 nausea. In addition, there were two paclitaxel dose reductions at DL2, one due to grade 3 fatigue and the second due to grade 3 abdominal pain.

### Efficacy

Among the ten patients included in the study, six (60%) patients had measurable disease and five (50%) of them had at least one tumor assessment after their enrolment on the study (one discontinued due to a DLT without a second tumor assessment after baseline). Two patients experienced a partial tumor response (both at DL1), two patients experienced stable disease (one per dose level) and one patient progressed at cycle 2. The two patients with partial tumor responses where on the study for 13.2 and 16.8 months.

### PK assessment

To assess the influence of the concomitant administration of paclitaxel and trastuzumab on dasatinib PKs, we compared dasatinib exposures alone (first PK occasion: day 2 of cycle 1) or combined (second PK occasion: day 18 on cycle 1).

**Figure 1:**
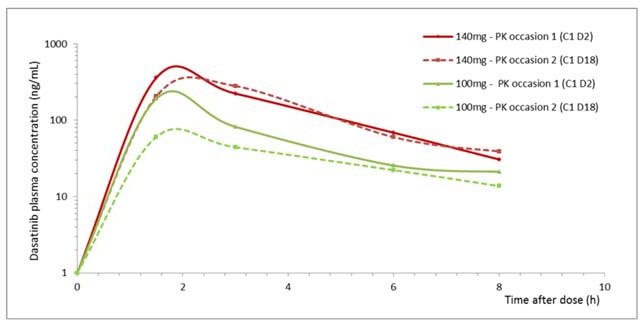
Mean profiles of dasatinib plasma concentration *versus* time sorted by dose and PK occasions in all treated patients

The mean profiles of dasatinib plasma concentrations versus time are shown in Figure 1, sorted by dose and PK occasion. The area under the plasma concentration-time curve from time zero to 8 hours post dose (AUC_0-8_) were calculated in all treated patients, as the dasatinib plasmatic half-life is very short and concentrations at 24 hours post dose could only be quantified in some patients.

Pk analysis was summarized in Table 4. The observed dasatinib mean half-life was low: 2.5 and 3.6 hours at DL1 and DL2, respectively on day 2 of cycle 1; and 3.4 and 2.4 on day 18 of cycle 1. The observed values of Cmax for DL1 were 191 and 78.4ng/mL on day 2 and 18 of cycle 1 and for DL2 were 422.3 and 314.4ng/mL, respectively. Similar findings were observed with AUC values, so paclitaxel and trastuzumab co-administration seems to slightly reduce dasatinib plasmatic exposure.

**Table 4:** Pharmacokinetic parameters of dasatinib and the combination with paclitaxel and trastuzumab

			t1/2 (h)		Cmax (ng/mL)		AUC (ng·h/mL)	
	n	Dasatinib dose (mg)	Mean	SD	Mean	SD	Mean	SD
Cycle 1 Day 2 Dasatinib	6	100	2,5	0,8	191,0	92,1	530,6	237,1
	3	140	3,6	1,4	422,3	56,4	1159,2	128,3
Cycle 1 Day 18 (Dasatinib+Paclitaxel+Trastuzumab)	4	100	3,4	1,7	78,4	51,1	248,1	156,7
	3	140	2,4	1,4	314,4	153,6	1047,3	682,8

t1/2: half life; h: hours; Cmax: maximum concentration; ng: nanogram; ml: milliliter; AUC: area under the curve; n: number of patients; SD: standard deviation.

### PD assessment

**Figure 2:**
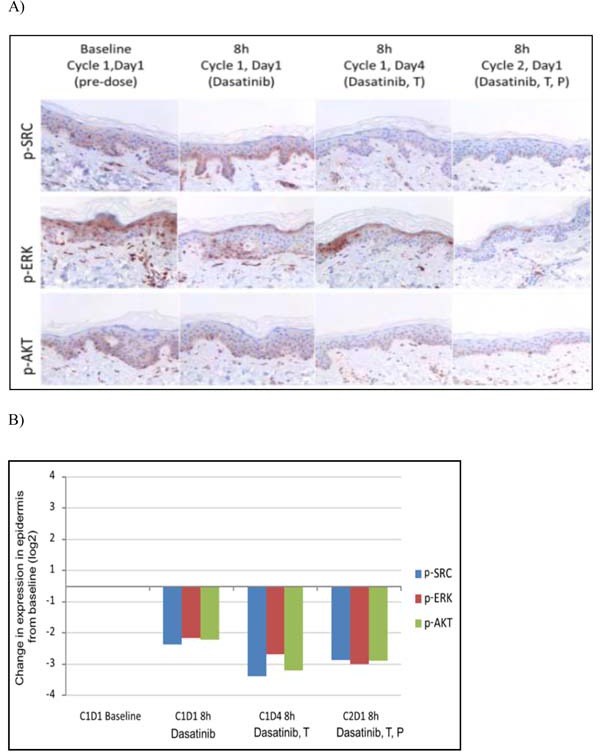
Immunohistochemical expression analysis of p-SRC, pAKT, p-ERK protein in sequential skin samples after treatment with dasatinib, the combination of dasatinib and trastuzumab (T) and in addition to paclitaxel (P) **A.** p-SRC was detected in the membrane of keratinocytes. Partial suprabasal layers of epidermis (C1D1) and complete (C1D4 and C2D1) downregulation in p-SRC expression was achieved after dasatinib treatment. p-ERK and p-AKT were evaluated in nucleus of epidermal cells, showing a similar pattern of inhibition. **B.** Bar graphs show differential expression from baseline of p-SRC, p-AKT, p-ERK along dasatinib treatment in combination with trastuzumab and paclitaxel.

Dasatinib alone induced a trend towards significant reduction in p-SRC keratinocyte expression on sequential skin biopsies (Mean baseline H-score ± 95% IC: 103 ± 13 vs. 28 ± 18 after 8h in C1D1, *p* = 0.066) (Figure 2). This reduction was maintained during the trastuzumab (C1D4) and paclitaxel (C2D1) administrations (Mean H-score ± 95% IC: 14 ± 10, *p* = 0.059; and 20 ± 12, *p* = 0.102, respectively). We observed a similar downregulation trend in p-ERK (Mean baseline H-score ± 95% IC: 63 ± 13 vs. 20 ± 9 in C1D1, *p* = 0.068; vs. 14 ± 10 in C1D4, *p* = 0.068; and vs. 11 ± 13 in C2D1, *p* = 0.109) and in p-AKT expression (Mean baseline H-score ± 95% IC: 65 ± 34 vs. 20 ± 10 in C1D1, *p* = 0.068; vs. 10 ± 0 in C1D4, *p* = 0.068; and vs. 13 ± 5 in C2D1, *p* = 0.109) (Figure 2). Treatments did not modify normal maturation of epidermis or induce dermal inflammatory infiltrates.

Finally, baseline total and phosphorylated markers on tumor samples were not associated with clinical efficacy due to the limited number of patients (data not shown).

## DISCUSSION

In the present article we demonstrate that the daily administration of dasatinib at 100 mg q.d. in combination with weekly paclitaxel 80 mg/m^2^ and trastuzumab 2 mg/kg is safe and have a good tolerability profile.

In our study, the RP2D of dasatinib in combination with weekly paclitaxel 80 mg/m^2^ and weekly trastuzumab 2 mg/kg was 100 mg q.d. We observed only one DLT at this dose level that was grade 3 transaminase elevation. Dasatinib at 140 mg q.d. exceeded the maximum tolerated dose, as two of the four treated patients experienced DLT (grade 3 pneumonitis and grade 3 nausea and vomiting not controlled with appropriate treatment).

At the RP2D, the toxicities were predictable and manageable. Grade 3 toxicities included, hyponatremia, fatigue, and transaminase elevation in a small proportion of patients (17%, *n* = 1, each), and diarrhea in 2 patients (33%). Grade 1 and 2 toxicities that appeared in at least the 50% of patients at this dose level included decrease of red blood cell count, anemia, neutropenia, leukopenia, lymphopenia, transaminase elevations, alkaline phosphate increase, hypocalcemia, nausea, oral mucositis, anorexia, fatigue, alopecia, arthralgia, and peripheral sensory neuropathy. This toxicity is in line with the use of taxanes in combination with trastuzumab [5, 6]. Therefore the addition of dasatinib to this regimen did not appear to add extra toxicities at the selected dose. The median number of cycles administered led to 12 with three patients receiving 14 or more cycles, what shows excellent long term tolerability.

We observed a moderate inter-patient variability of dasatinib clearance when combined with paclitaxel and trastuzumab. These differences could be due to the concomitant administration of CYP inducers, such as corticoids, which are used prior to paclitaxel administration to avoid infusion reactions. However, this modification in the clearance of dasatinib seems not to be translated into unexpected toxicities or limit the pharmacodynamic effect of dasatinib on selected biomarkers.

This study was focused in dasatinib pharmacokinetic, but its influence on the metabolism of the others drugs was not evaluated. Dasatinib is a time-dependent weak inhibitor of CYP3A4, so dasatinib may have influence on the metabolism of other CYP3A4 substrate such as paclitaxel. However, studies published in the literature reported that paclitaxel exposure was unchanged by concurrent administration of dasatinib. Secord et al [24] published results of a phase I trial and reported that co-administration of dasatinib and paclitaxel did not significantly alter either dasatinib or paclitaxel exposure.

We identified a reduction of p-ERK, p-SRC and p-AKT expression in keratinocytes of the epidermis after dasatinib treatment, which was further reduced when trastuzumab was added. These findings confirm the mechanism of action identified in preclinical models, where the combination of dasatinib and trastuzumab reduced the phosphorylated levels of AKT, ERK and SRC [23, 25, 26]. Our group reported preclinical data showing that the addition of dasatinib to trastuzumab increased the antitumoral activity of trastuzumab through the combined inhibition of pAKT and pSRC [23]; and dasatinib exerted an inhibitory effect on pERK [25]. In addition, this combination was synergistic. The results obtained in this study confirm those preclinical findings [23].

In conclusion, in this report we describe the feasibility of the combination of dasatinib, trastuzumab and paclitaxel, observing an optimal toxicity profile, and the proof of concept of its synergistic effect on proteins involved in trastuzumab resistance. The phase II part of this study is currently evaluating efficacy and has recently finished recruitment. The results will be reported soon.

## MATERIAL AND METHODS

### Eligibility

Patients with HER2-overexpressing metastatic breast cancer, confirmed by a central reference laboratory, were eligible if they fulfilled the following criteria: women aged ≥18 years; Eastern Cooperative Oncology Group (ECOG) performance status ≤1; no prior chemotherapy or anti-HER2 therapy for metastatic breast cancer (patients treated with adjuvant chemotherapy regimens based on taxanes or anti-HER2 therapies, including but not limited to trastuzumab or lapatinib, were allowed to be enrolled on the study if at least 12 months had elapsed from the end of these treatments); adequate bone marrow, liver, and renal functions; normal cardiac function, and no concurrent medical condition that may increase the risk of toxicity. Patients with central nervous system (CNS) metastases were allowed if treated and clinically stable without medication. Patients may have measurable or non-measurable disease. Written informed consent was obtained and documented before performing any protocol-specific procedure. The study was conducted in accordance with the International Conference on Harmonization Good Clinical Practice Guidelines (ICH GCP), the Declaration of Helsinki and applicable local regulatory requirements and laws. The protocol was approved by the Institutional Review Board and the Ethics Committee of the participating sites, according to the requirements of the Spanish regulations (GEICAM/2010-04; clinicaltrials.gov identifier: NCT01306942).

### Study design and treatment

The trial was an investigator-initiated study sponsored and coordinated by GEICAM. This was a single-arm, open-label, multicenter, phase I-II study. The primary objective for the phase I part of the study was to determine the maximum tolerated dose (MTD) and recommended phase II dose (RP2D) of dasatinib in combination with fixed doses of trastuzumab and paclitaxel. The secondary objectives included the efficacy, measured by the objective response rate (ORR) according to the Response Evaluation Criteria in Solid Tumors (RECIST) version 1.1, as well as the safety, pharmacokinetics (PKs) and pharmacodynamics (PDs) of the combination.

A “3+3” design guided dose escalation was used for this trial, and no intra-patient escalation was permitted. All patients in each dose level must had completed at least the first cycle of therapy before allowing the inclusion of patients in the next dose level. The recommended phase II dose (RP2D) was defined as the dose in which ≤1 out of 6 patients developed a dose limiting toxicity (DLT). DLT was defined as an adverse event (AE) at least possibly related to the study medications during the first treatment cycle. AEs considered as DLTs included those needing any dose modification due to toxicity within the first cycle; grade 4 neutropenia lasting ≥7 days; grade 4 thrombocytopenia or grade 3 complicated with bleeding; grade 3 or 4 neutropenia associated with fever ≥38.5°C or infection; grade 3 or 4 non-hematological toxicity—excluding alopecia or sub-optimally managed nausea, vomiting or diarrhea—or any toxicity resulting in inability to initiate the second cycle within 14 days of the end of cycle 1. Paclitaxel and trastuzumab were administered at a fixed dose in 4-week cycles: weekly trastuzumab 2mg/kg intravenously (IV), following a loading dose of 4mg/kg in cycle 1 and weekly paclitaxel (80 mg/m^2^) for three weeks followed by a rest period of 1 week. Oral dasatinib was administered q.d. (once daily) at two dose levels: 100mg (DL1) and 140 mg (DL2). The first cycle had duration of 38 days in all patients included at each dose level. In this cycle Dasatinib was initiated on D1 and trastuzumab was added on D4. Paclitaxel was added on D11. Dose modifications were permitted after the first cycle as indicated in the protocol. Treatment was continued until radiographic or symptomatic progression, or unacceptable toxicity occurred, or withdrawal of the informed consent. Patients with permanent discontinuation of any of the study drugs were discontinued from the study.

### Safety and efficacy assessment

Safety was assessed using the National Cancer Institute (NCI) Common Terminology Criteria for Adverse Events (CTCAE) version 4.03 and the worst AE per cycle was reported. Efficacy was evaluated every two cycles (eight weeks) according to the RECIST, version 1.1. Patients discontinuing the study treatment for a reason other than disease progression were followed until objective progression was observed.

### PK assessment

PK sampling was assessed at six time points (before and 1.5, 3, 6, 8 and 24 hours (h) after dasatinib administration) on days (D) 2, 18 and 32 of the first cycle and on D1 of the second and third cycles. After protocol amendment, PK samples on the second and third cycles were reduced and were obtained only at three points: before, 6 and 8 h post-administration.

Blood samples were collected into heparin containing tubes and were quickly centrifuged at 3000 x g for 10 minutes at 4°C, aliquoted and stored at −70°C until analysis. Dasatinib and paclitaxel plasma concentrations were determined by validated high-performance liquid chromatography with ultraviolet-light detection (HPLC-UV), and trastuzumab plasma concentrations were determined by an immunoassay, using the enzyme-linked immunosorbent assay (ELISA) method.

PK parameters were estimated by non-compartmental model trapezoidal approach using Phoenix^®^ WinNonlin^®^ software, version 7.2.

### PD assessment

Immunohistochemistry (IHC) expression analysis of phosphorylated (p) p-SRC, p-ERK, p-AKT and total SRC, ERK and AKT proteins were performed in Formalin-Fixed Paraffin-Embedded (FFPE) sequential skin samples at three time points on cycle (C) 1 (D1, before and 8 h after dasatinib administration and D 4, 8 h post-treatment), and one time point on C 2 (D 1, 8 h post-treatment). Same analysis was determined in baseline FFPE tumor samples. The antibodies used were: anti-SRC (rabbit monoclonal SRC antibody clone 36D10, CST, Beverly, MA), anti-p-SRC (rabbit polyclonal SRC Tyr527 antibody, CST), anti-ERK1/2 (rabbit monoclonal p44/42 MAPK antibody clone 137F5, CST), anti-p-ERK1/2 (rabbit monoclonal phospho-p44/42 MAPK Thr202/Tyr204 antibody clone D13.14.4E, CST), anti-protein kinase AKT (rabbit monoclonal antibody clone 11E7, CST) and anti-p-protein kinase AKT (rabbit monoclonal AKT Ser473 antibody clone D9E, CST). All assays were determined on 4 μm tissue sections by immunohistochemistry using a Dako Link Autostainer. Heat antigen retrieval was carried out in pH 9 EDTA-based buffered solution in a Dako Link platform. Endogenous peroxidase was quenched. Primary antibody-antigen reaction was detected by incubation with appropriate polymers coupled with peroxidase (Flex+; Dako). Sections were visualized with 3,3#-diaminobenzidine (DAB) and counterstained with Hematoxylin. Same sections incubated with non-immunized serum were used as negative controls. As positive control, sections of human tumors with known marker expression were used.

Expression of markers was assessed in a blinded fashion by an experienced pathologist. For ERK1/2, p-ERK1/2, AKT and p-AKT, nuclear staining was required for considering a cell as positive. SRC and p-SRC were evaluated as membranous and cytoplasmic staining in target cells. A semiquantitative histoscore (H-score) was calculated for all markers. The H-score was determined by estimation of the percentage of tumor cells positively stained with low, medium, or high staining intensity. The final score was determined after applying a weighting factor to each estimate. The formula used was H-score = (low %) × 1 + (medium %) × 2 + (high %) × 3, and the results ranged from 0 to 300.

Biomarker changes in sequential skin biopsies were statistically analyzed by Wilcoxon rank sum test. In addition, biomarker basal expression in tumor and clinical response was correlated by Univariate Logistic Regression. SPSS (v21.0) and SAS Enterprise Guide v5.1 was used for all analysis, considering significance in P values of less than 0.05.
